# The Paradox of Inclusion: Older Adults With Learning Disabilities in Policy and Practice

**DOI:** 10.1093/geroni/igaf122.4215

**Published:** 2025-12-31

**Authors:** Anastasiia Brus, Judith Sixsmith, Joe Tai, Darren Chadwick, Mei Lan Fang

**Affiliations:** Ukrainian Catholic University, Lviv, L’vivs’ka Oblast’, Ukraine; University of Dundee, Dundee, Scotland, United Kingdom; University of Dundee, Dundee, Scotland, United Kingdom; Liverpool John Moores University, Liverpool, England, United Kingdom; Simon Fraser University, Vancouver, British Columbia, Canada

## Abstract

As part of the UK IncludeAge Study, we explore how policy, service structures, and organisational practices shape opportunities for the inclusion of older adults with learning disabilities. Despite policy frameworks promoting integration, significant barriers persist that paradoxically reinforce exclusion. The aim was to examine these tensions and identify pathways for systemic change. Our approach involved conducting ten semi-structured interviews with representatives from local Older Adult Social Care departments (n = 5) and voluntary or charitable organisations (n = 5) across England and Scotland. Data were analysed using Reflexive Thematic Analysis to identify recurring themes and contradictions in organisational approaches to inclusion. Substantial and intersecting barriers were identified. The accessibility of information remains a major challenge, with complex language, bureaucratic procedures, and poor dissemination limiting access to services. Tools intended to foster inclusion often perpetuated exclusion. Staff reluctance to address sensitive issues created silences around important areas of life, while paternalistic attitudes framed older adults with learning disabilities as “child-like.” Though intended as protection, such practices restricted autonomy and increased dependency. Policy programmes were frequently designed without the involvement of older adults with learning disabilities, resulting in fragmented provision and the need for retrospective “inclusive” fixes. Limited facilitators, such as staff training, voluntary sector initiatives, and community collaborations, were inconsistent and resource-dependent. Findings reveal a paradox of inclusion: older adults with learning disabilities are selectively recognised, experiencing conditional rather than full citizenship. Meaningful change requires embedding accessibility, dignity, and identity at the policy-making stage to ensure genuine participation in community life.

